# Diagnostic and Prognostic Significance of a Four-miRNA Signature in Colorectal Cancer

**DOI:** 10.3390/ijms26031219

**Published:** 2025-01-30

**Authors:** Giuseppe Gattuso, Federica Longo, Graziana Spoto, Daria Ricci, Alessandro Lavoro, Saverio Candido, Antonio Di Cataldo, Giuseppe Broggi, Lucia Salvatorelli, Gaetano Magro, Massimo Libra, Luca Falzone

**Affiliations:** 1 Department of Biomedical and Biotechnological Sciences, University of Catania, 95123 Catania, Italy; peppeg9305@gmail.com (G.G.); fedelongo08@gmail.com (F.L.); grazianaspoto@gmail.com (G.S.); daria95.ricci@gmail.com (D.R.); alessandrolavoro@ymail.com (A.L.); scandido@unict.it (S.C.); 2 Research Center for Prevention, Diagnosis and Treatment of Cancer, University of Catania, 95123 Catania, Italy; 3 Department of Medical and Surgical Sciences and Advanced Technologies “G.F. Ingrassia”, University of Catania, 95123 Catania, Italy; adicataldo@unict.it (A.D.C.); giuseppe.broggi@unict.it (G.B.); lucia.salvatorelli@unict.it (L.S.); gaetano.magro@unict.it (G.M.)

**Keywords:** microRNAs, colorectal cancer, epigenetics, biomarkers, droplet digital PCR, bioinformatics

## Abstract

Colorectal cancer (CRC) is the fourth most commonly diagnosed cancer and one of the leading causes of cancer death worldwide. Despite diagnostic and therapeutic advances, CRC mortality remains high, especially in industrialized countries. Numerous studies have highlighted the pathogenetic role of altered microRNA (miRNA) expression among the various factors contributing to the development and progression of colorectal cancer (CRC). However, the data regarding specific miRNAs involved in CRC pathogenesis remain inconsistent, and no miRNAs have been recognized so far as reliable or effective biomarkers for the diagnosis of this tumor type. To identify novel miRNA biomarkers in CRC, this study validated the expression levels of a four-miRNA signature predicted to be involved in CRC by analyzing both tissue and liquid biopsy samples. Our experimental and bioinformatics results highlighted the diagnostic potential of hsa-miR-21-5p, hsa-miR-503-5p, and hsa-miR-375, as well as the potential prognostic value of hsa-miR-497-5p overexpression and hsa-miR-375-3p downregulation. Overall, the results obtained suggest the diagnostic and prognostic significance of this four-miRNA signature in CRC.

## 1. Introduction

Colorectal cancer (CRC) is one of the most frequently diagnosed tumors in the world. According to the latest age-standardized epidemiological data, CRC represents the third most prevalent type of cancer in both sexes after lung cancer and breast cancer. It is also the second leading cause of cancer-related death and is more frequently diagnosed in males than in females. Furthermore, the incidence of CRC appears to be correlated with geographical distribution, with Asia and Europe recognized as the geographical areas with the highest incidence rates [1]. As demonstrated by these epidemiological data, CRC is still one of the tumors with the highest number of deaths per year despite advances in diagnostic and therapeutic strategies. However, the implementation of screening programs has contributed to a reduction in mortality by enabling the early detection of tumors, which allows for timely and more effective therapeutic interventions, ultimately improving patients’ prognosis [2,3]. To further enhance these outcomes, it is crucial to strengthen and expand these screening programs. The most common screening tests for CRC include invasive approaches such as sigmoidoscopy, colonoscopy, and colonoscopy associated with computed tomography, as well as non-invasive strategies like the fecal occult blood test (FOBT), fecal immunochemical test (FIT), fecal DNA test, and double-contrast barium enema [4].

Several biomarkers have also been proposed for the early diagnosis and monitoring of CRC. For instance, CEA and CA19-9 are biomarkers with a good predictive value for disease monitoring but have low specificity and sensitivity for the diagnosis of CRC [5,6]. Despite all these screening methods, the gold standard method for CRC diagnosis is still represented by the histopathological examination of a suspected lesion identified after surgical resection of the colon or during colonoscopy.

In this scenario, epigenetic alterations have recently been proposed as novel promising biomarkers for CRC diagnosis [7,8]. Among these, alterations in microRNA (miRNA) expression levels are of particular diagnostic significance. Notably, miRNAs are small non-coding RNAs ranging from approximately 19 to 25 nucleotides in length that play a key role in the regulation of gene expression [9]. To date, it is well known that miRNAs are involved in the initiation and progression of several types of cancer, including brain [10], lung [11], breast [12], oral [13], prostate [14], melanoma [15], and colorectal [16]. Through a mechanism known as messenger RNA interference (RNAi), miRNAs target specific mRNAs commonly interacting with their 3′-UTR and influencing its expression. Furthermore, miRNAs can be secreted into circulation and persist in remarkably stable forms [17,18].

From a pathogenetic point of view, miRNAs can be divided into two main categories based on their molecular targets: tumor suppressor miRNAs and oncogenic miRNAs. Therefore, miRNAs can regulate some cellular and molecular pathways associated with the development and progression of tumors, including cell proliferation and migration, autophagy, apoptosis, epithelial-mesenchymal transition (EMT), and drug resistance [19]. Since miRNA expression is profoundly dysregulated in the presence of specific diseases, they have been proposed as potential tumor biomarkers.

Regarding CRC, several studies have analyzed miRNA expression in CRC tissues versus adjacent non-cancerous tissues to identify potential biomarkers for this tumor [20,21,22,23]. Similarly, other studies investigated the circulating levels of miRNAs in liquid biopsy samples obtained from CRC patients and healthy donors to propose non-invasive strategies for the detection of CRC [24,25]. Among these studies, our research group previously conducted an integrated computational analysis considering all the miRNA microarray expression datasets related to CRC and identified a list of 20 miRNAs frequently dysregulated in CRC. Of these miRNAs, 10 were upregulated (hsa-miR-1246, hsa-miR-1308, hsa-miR-135b-5p, hsa-miR-183-5p, hsa-miR-18a-5p, hsa-miR-18b-5p, hsa-miR-21-5p, hsa-miR-223-3p, hsa-miR-224-5p, and hsa-miR-503-5p) and 10 were downregulated (hsa-miR-1-3p, hsa-miR-133b, hsa-miR-143-3p, hsa-miR-145-5p, hsa-miR-150-5p, hsa-miR-195-5p, hsa-miR-215-5p, hsa-miR-375, hsa-miR-378-3p, and hsa-miR-497-5p) [26]. Based on our previous results and data from the literature, we selected four miRNAs strongly dysregulated in CRC, of which two were upregulated (hsa-miR-21-5p and hsa-miR-503-5p) and two were downregulated (hsa-miR-375 and hsa-miR-497-5p). These miRNAs were selected to perform validation analyses on CRC formalin-fixed paraffin-embedded (FFPE) tissue samples and liquid biopsy samples obtained from CRC patients and healthy donors to propose a novel predictive miRNA signature for CRC. To obtain more reliable results, miRNAs were quantified using the droplet digital PCR (ddPCR) assay, which demonstrated greater accuracy when used for the analysis of critical samples like FFPE tissue samples and liquid biopsy samples where the target is highly fragmented or low-represented [27,28,29].

## 2. Results

### 2.1. Clinical Validation of the Diagnostic Role of a Four-miRNA Signature in Colorectal Cancer Samples

**Figure 1:**
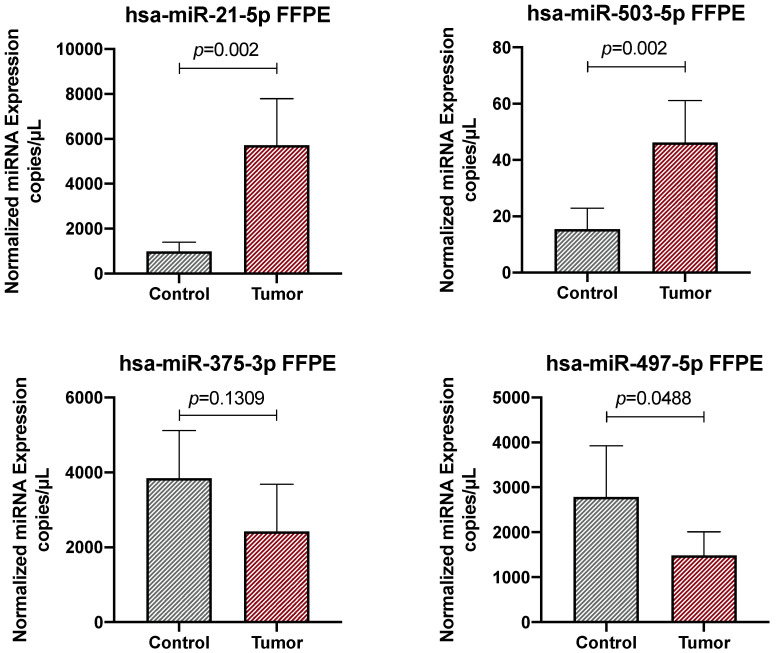
Absolute quantification of the expression levels of hsa-miR-21-5p, hsa-miR-503-5p, hsa-miR-375-3p, and hsa-miR-497-5p in FFPE CRC and adjacent normal mucosa samples. Results are expressed as copies/μL of the ddPCR reaction. The data were considered statistically significant for *p* < 0.05.

In order to investigate the diagnostic potential of the four selected miRNAs, the expression levels of hsa-miR-21-5p, hsa-miR-503-5p, hsa-miR-375-3p, and hsa-miR-497-5p were analyzed in both tumor and normal adjacent FFPE tissue samples. The analysis of the miRNA expression levels according to patients’ socio-demographic and clinical-pathological features revealed no statistically significant differences when considering age, gender, smoke status, tumor stage, and the presence of positive lymph nodes (Supplementary Table S1). The ddPCR results revealed statistically significant differences between the expression levels of almost all the selected miRNAs in the tumor samples vs normal control, thus confirming the results of our previous computational analyses [26]. As expected, the expression levels of the two predicted upregulated miRNAs, hsa-miR-21-5p and hsa-miR-503-5p, were found to be significantly upregulated in CRC tissues compared to normal adjacent controls (*p* = 0.002) (Figure 1). Similarly, the expression level of the downregulated miRNA hsa-miR-497-5p was also significantly lower in CRC tissue samples compared to normal tissues (*p* = 0.0488), while a non-significant trend of reduction was observed for hsa-miR-375-3p in the tumor samples (*p* = 0.1309) (Figure 1).

**Figure 2:**
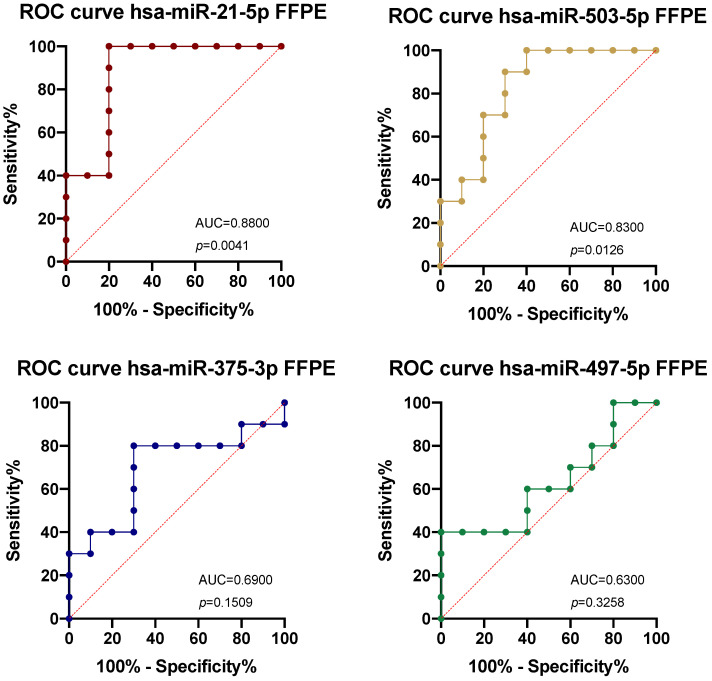
ROC curve analysis of hsa-miR-21-5p, hsa-miR-503-5p, hsa-miR-375-3p, and hsa-miR-497-5p expression levels obtained in FFPE samples. The data were considered statistically significant for *p* < 0.05.

Overall, the data obtained for the FFPE tissue samples confirmed the bioinformatic results, suggesting that the assessment of the expression levels of the selected miRNAs in the CRC biopsies could also be indicative of the presence of the tumor in suspicious pre-neoplastic lesions. ROC analyses further confirmed the diagnostic potential of the selected miRNAs, demonstrating excellent diagnostic accuracy for the two upregulated miRNAs hsa-miR-21-5p and hsa-miR-503-5p with AUC values of 0.88 (*p* = 0.0041) and 0.83 (*p* = 0.0126), respectively (Figure 2). With regards to the two downregulated miRNAs, none of these miRNAs can be considered diagnostic biomarkers due to the low and non-significant AUC values obtained (Figure 2).

For the four selected miRNAs, the sensitivity and specificity values obtained are reported in Table 1.

**Figure 3:**
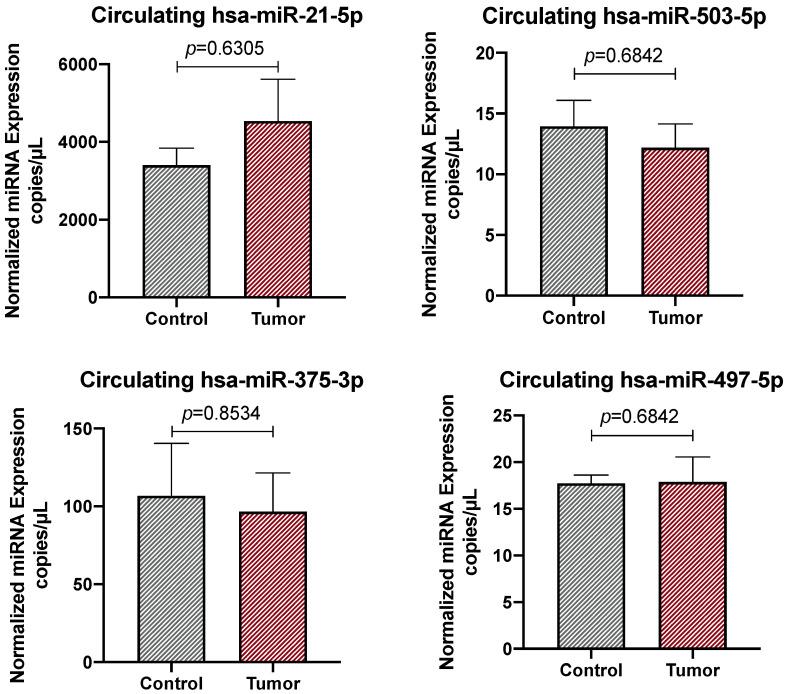
Absolute quantification of the expression levels of hsa-miR-21-5p, hsa-miR-503-5p, hsa-miR-375-3p, and hsa-miR-497-5p in liquid biopsy samples obtained from CRC patients and healthy donors. Results are expressed as copies/μL of the ddPCR reaction. The data were considered statistically significant for *p* < 0.05.

To propose these four miRNAs as circulating biomarkers for the early diagnosis of CRC and the development of non-invasive screening strategies for individuals at risk of this cancer, the expression levels of the four selected miRNAs were evaluated in liquid biopsy samples obtained from CRC patients and healthy donors by using the same ddPCR platform. The results obtained from these analyses were less convincing than those obtained in FFPE samples. Indeed, the ddPCR absolute quantification of the selected miRNAs showed only a non-significant trend of increment for the predicted upregulated hsa-miR-21-5p and a decrement for the predicted downregulated hsa-miR-375-3p. No trends were observed for hsa-miR-503-5p and hsa-miR-497-5p, probably due to the limited sample size of our case series (Figure 3).

Since no significant data were obtained for circulating miRNAs, no ROC analyses were performed for these data. Overall, the less significant results obtained by analyzing liquid biopsy samples may be also due to the dilution of CRC-related miRNAs with miRNAs deriving from other tissues and organs; therefore, further investigations are needed to isolate and evaluate only circulating tumor-related miRNAs (29). Nonetheless, the ddPCR results obtained from the FFPE samples confirmed the good predictive value of the computational analyses previously performed and the potential clinical utility of miRNAs for the early diagnosis of CRC, while further and in-depth investigations are needed for circulating miRNAs.

### 2.2. Bioinformatics Evaluation of the Prognostic Value of the Four-miRNA Signature

**Figure 4:**
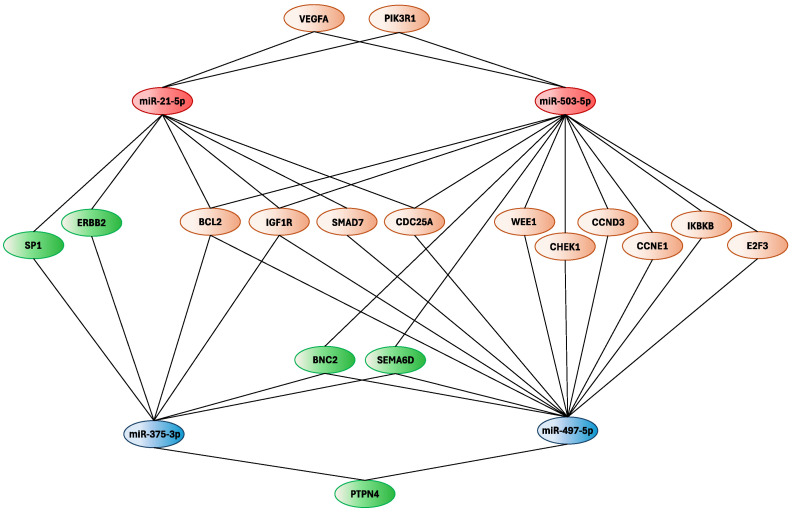
Genes targeted by the four-miRNA signature according to miRTargetLink Human, miRDip, TargetScan, and miRWalk software. The targeted genes identified by using miRDip, TargetScan, and miRWalk are displayed in green, while the common miRNA-targeted genes identified through miRTargetLink Human 2.0 are displayed in orange.

By consulting the miRTargetLink Human software version 2.0, genes directly modulated by the four selected miRNAs were identified. Specifically, by using different versions of the miRTargetLink Human tool and confirming these data with those obtained from miRDip, TargetScan, and miRWalk, the genes targeted by at least two different miRNAs were identified. Specifically, the selected miRNAs could alter the expression levels of a total of 17 different genes with strong levels of interaction, of which *BLC2* and *IGF1R* showed the highest number of interactions (Figure 4).

Notably, *BCL2*, *IGF1R*, *SMAD7*, and *CDC25A* are involved in neoplastic transformation, therefore, their dysregulation, mediated by altered miRNA expression levels, may be responsible for CRC development. Regarding hsa-miR-497-5p and hsa-miR-503-5p, the common targets were *E2F3*, *CCND3*, *CHEK1*, *WEE1*, *IKBKB*, and *CCNE1*, all of which are genes involved in cell cycle regulation. hsa-miR-21-5p and hsa-miR-503-5p showed strong interactions with *PIK3R1* and the *VEGFA*, while *PTPN4* was the only target commonly modulated by the two downregulated miRNAs. Finally, the other two genes identified through this analysis were ERBB2 and SP1, known to be involved in CRC progression, and modulated by hsa-miR-21-5p and hsa-miR-375-3p.

**Figure 5:**
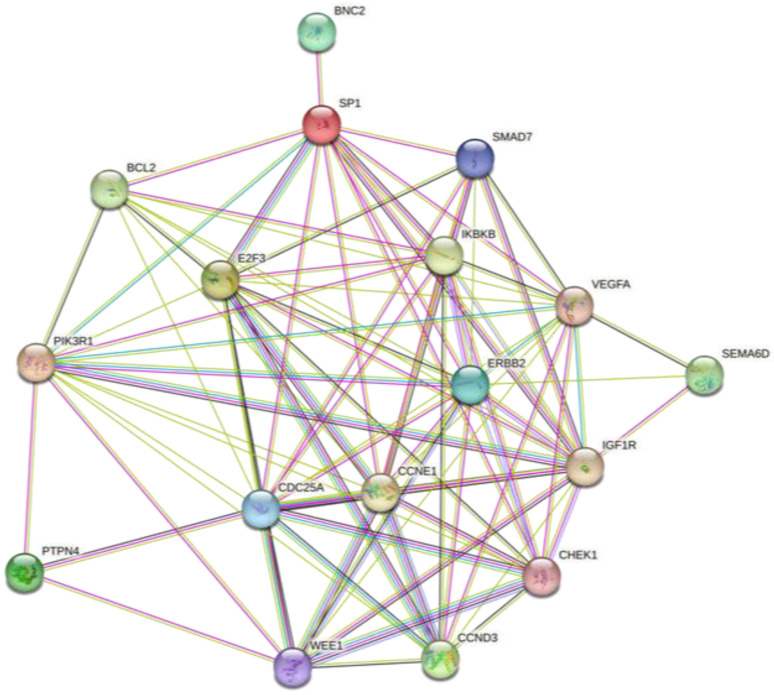
Protein-protein interaction network between the genes strongly modulated by the four validated miRNAs according to STRING analysis (version 11.5, confidence value 0.150).

To better elucidate the functional interaction of these 17 miRNA-targeted genes, a protein-protein interaction analysis was performed using the STRING tool (Figure 5).

All the miRNA-targeted genes showed a strong protein interaction network with better results obtained for the genes involved in cell cycle regulation like *WEE1*, *CHEK1*, *CDC25A*, *CCND3*, *IGF1R*, and *CCNE1*.

Subsequently, a GO Panther analysis was performed to assess the involvement of these genes in the modulation of biological processes, molecular functions, protein classes, and molecular pathways potentially associated with CRC development and progression.

**Figure 6:**
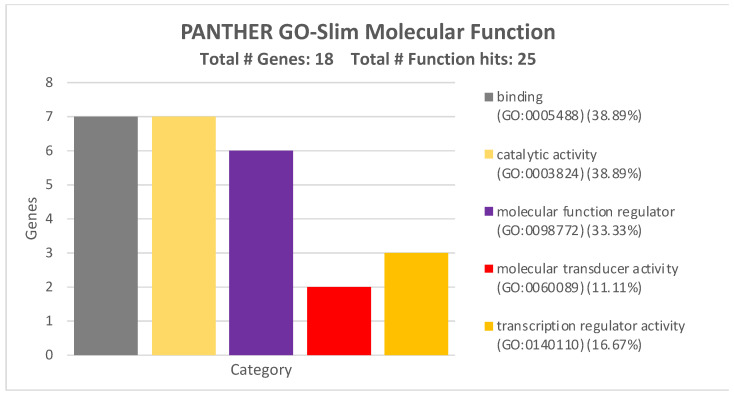
Molecular functions of the 17 genes strongly modulated by the four validated miRNAs according to GO PANTHER analysis.

With regards to the molecular functions, the four miRNAs and their 17 targeted genes were involved in the modulation of five major molecular functions, i.e., binding activity, catalytic activity, molecular function regulator activity, molecular transducer activity, and transcription regulator activity. Within all these functions, these genes regulated a plethora of downstream mediators involved in CRC development (Figure 6).

**Figure 7:**
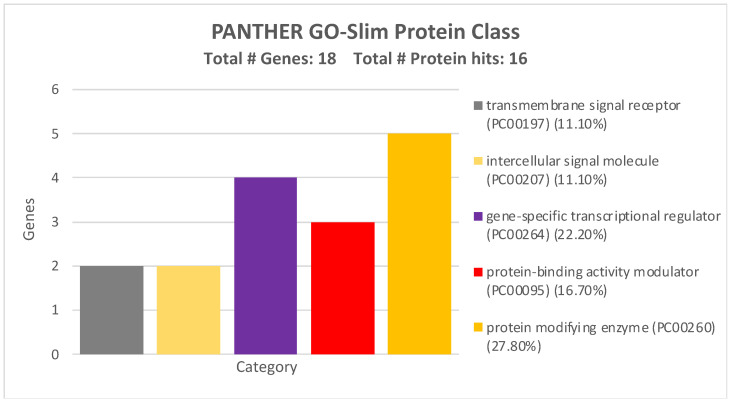
Protein classes of the 17 genes strongly modulated by the four validated miRNAs according to GO PANTHER analysis.

By analyzing the protein classes of the miRNA-targeted genes, another five groups were identified: in further detail, five genes were protein modifying enzymes; three were protein-binding activity modulators; two were transmembrane signal receptors; four were gene-specific transcriptional regulators; and two were intercellular signal molecules, suggesting how the miRNAs were able to induce CRC by epigenetically modulating several determinants of different cell compartments (Figure 7).

**Figure 8:**
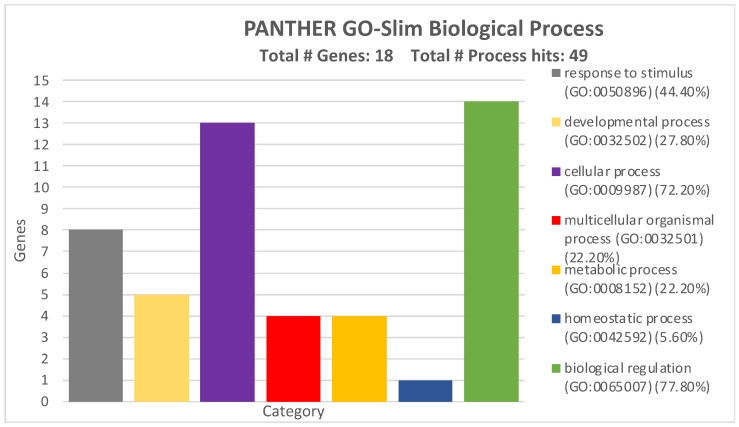
Biological processes of the 17 genes strongly modulated by the four validated miRNAs according to GO PANTHER analysis.

Subsequently, the involvement of the selected miRNA-targeted genes in the regulation of biological processes was investigated. As displayed in Figure 8, the 17 genes were involved in a wide variety of biological processes, among which the biological regulation, cellular, and metabolic processes were the most represented. Response to stimuli and developmental processes were also widely modulated by miRNAs and their targeted genes (Figure 8).

**Figure 9:**
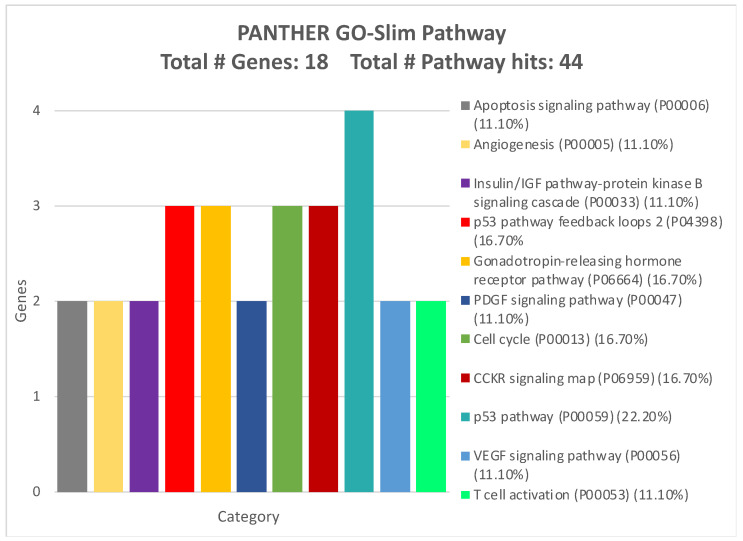
Molecular pathways of the 17 genes strongly modulated by the four validated miRNAs according to GO PANTHER analysis (only pathways showing two genes were reported).

Figure 9 shows the association between the 17 highlighted genes and the pathways in which they are involved. The majority of the genes were involved in cell cycle-related pathways, p53 pathways, the gonadotropin-releasing pathway, and the CCKR signaling map, thus confirming the results also obtained by using the STRING tool (Figure 9).

All the GO Panther analyses revealed how the four miRNAs can alter a wide variety of pathways and cellular-molecular processes by modulating the expression levels of these 17 genes.

Since the primary mechanism of action of the miRNAs was the modulation of gene expression, we then evaluated the expression levels of these 17 miRNA-targeted genes in the cohort of CRC patients obtained from the TCGA database by using the GEPIA portal. In addition, OncoLnc was used to evaluate the prognostic significance of the selected miRNAs. Both bioinformatics tools employed the data deposited in the TCGA COAD and READ databases and allowed a more detailed study into the roles of the selected miRNAs and their respective targets in CRC.

**Figure 10:**
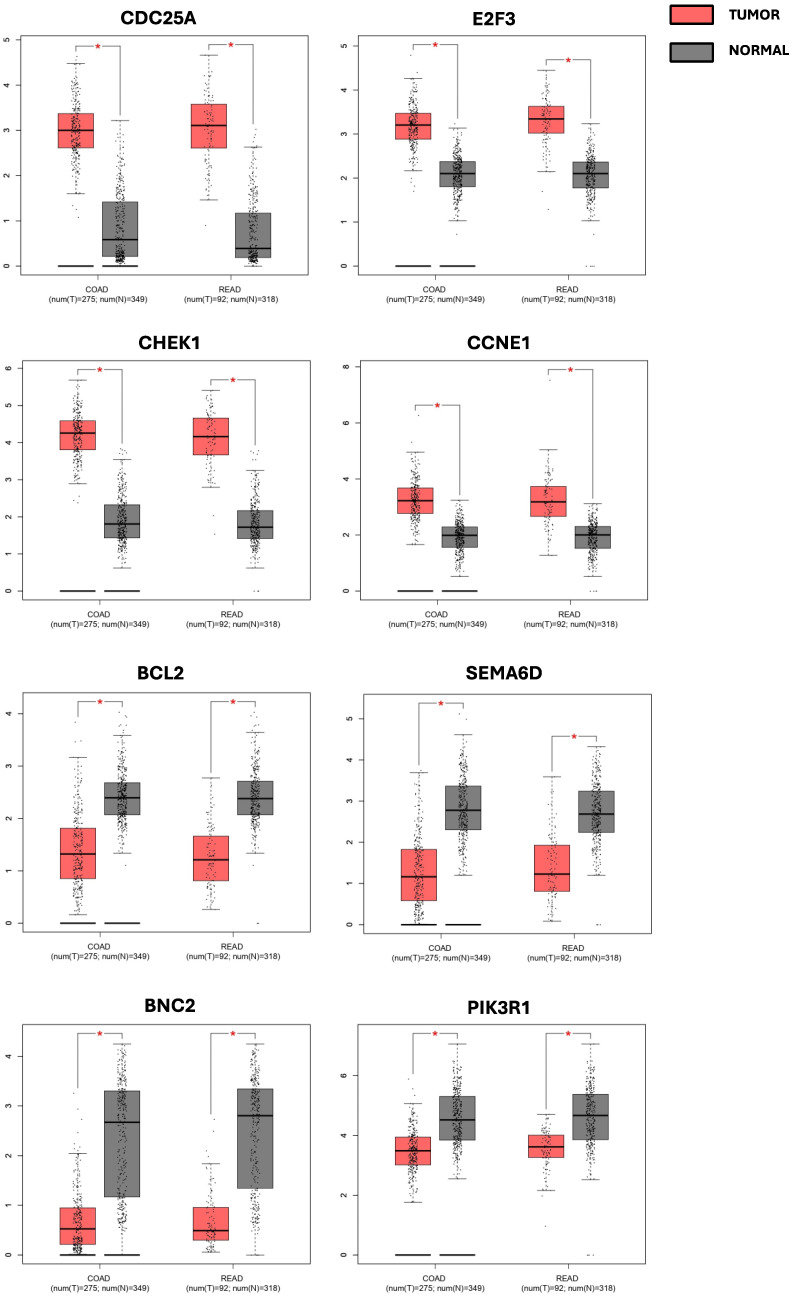
GEPIA analysis of the expression levels of the miRNA-targeted genes in COAD and READ patients compared with healthy controls, according to data from the TCGA and GTEx databases. The *p*-value threshold was set at 0.01 (* = *p* < 0.01). The relative expression levels were first log2(TPM+1) transformed and the log2FC was defined as median (Tumor)—Median (Normal), where TPM is the transcript count per million.

The GEPIA results showed that eight out of the seventeen targeted genes presented dysregulated expression levels in both COAD and READ databases (Figure 10).

Specifically, the GEPIA analysis highlighted that *BCL2*, *BNC2*, *SEMAD6*, and *PIK3R1* expression were downregulated in the COAD and READ databases compared to the healthy controls contained in the GTEx database. *CHEK1*, *CDC25A*, *CCNE1*, and *E2F3* showed a significantly higher expression in both COAD and READ patients compared to healthy controls. Finally, *ERBB2* expression levels followed a different pattern: a slight increase was observed for READ patients, but not for COAD ones.

**Figure 11:**
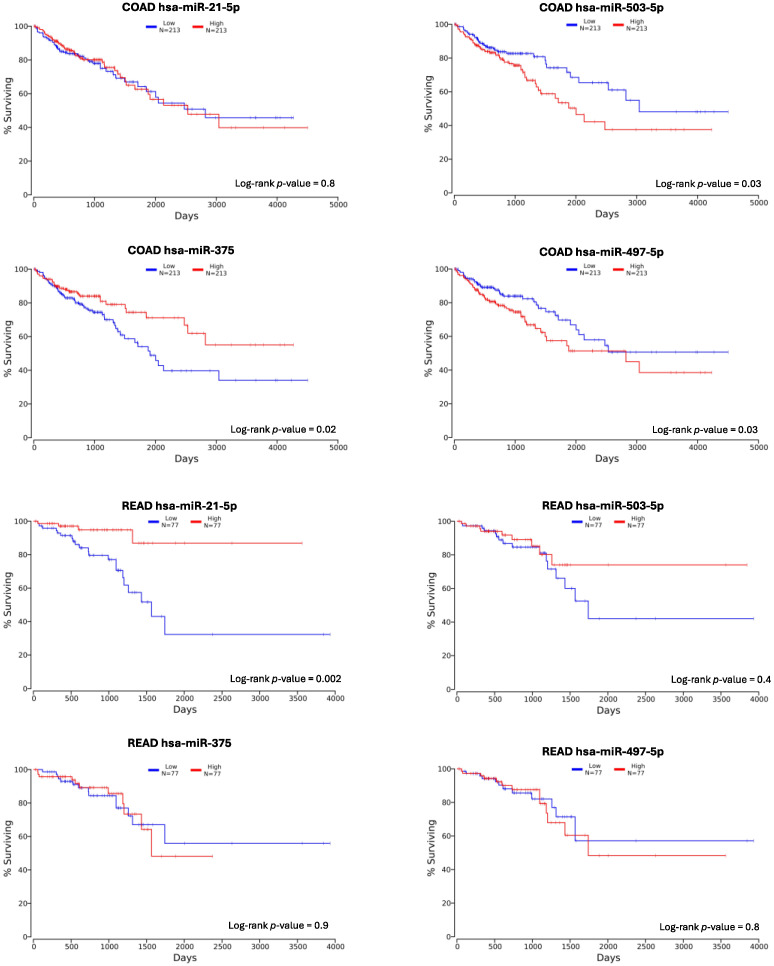
OncoLnc analysis of the overall survival (OS) rates of the COAD and READ patients according to the expression levels of the four selected and validated miRNAs. Log-rank *p*-values < 0.05 were considered statistically significant.

In order to computationally assess the possible prognostic role of the four selected and validated miRNAs, the OncoLnc tool was consulted. The results of this analysis, performed on the data obtained from the TCGA COAD and READ databases, revealed that the dysregulation of these miRNAs correlated with poor prognosis (Figure 11).

In particular, no significant data were obtained for hsa-miR-21-5p in the COAD database analysis, whereas using the READ database, we found a significant prognostic value for this miRNA (*p*-value = 0.002). However, contrary to what was expected, the worse prognosis for patients correlated with a lower expression of hsa-miR-21-5p. Concerning the hsa-miR-503-5p, discordant data were found between the COAD and READ databases. Specifically, while for READ there was a trend of better survival when this miRNA was overexpressed, for COAD patients, the overexpression of hsa-miR-503-5p correlated with a worse prognosis as predicted by the bioinformatics analyses. The predicted and validated downregulated hsa-miR-497-5p seemed to correlate with worse prognosis when overexpressed in COAD patients, however, no clear survival association was obtained for this miRNA. Regarding hsa-miR-375-3p, the survival graphs showed a positive correlation between high miRNA levels and better prognosis in COAD patients, while no association was found for READ patients.

**Figure 12:**
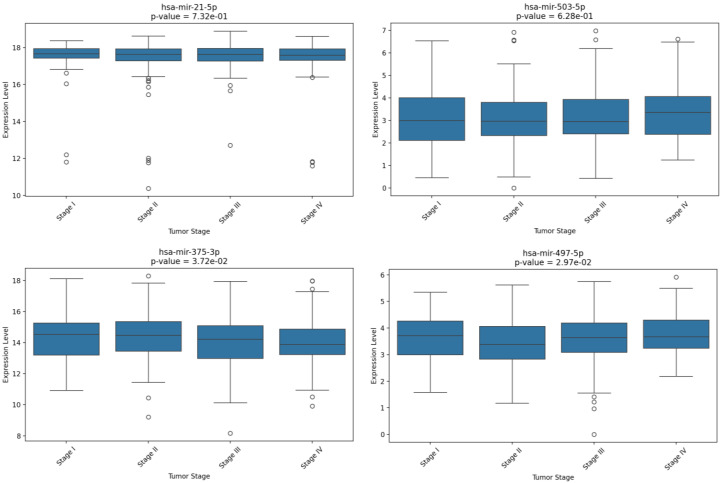
Expression levels of the four selected and validated miRNAs in CRC samples at various stages of progression (Stage I to Stage IV). The data were considered statistically significant for *p* < 0.05.

Finally, the analysis of the expression levels of the four-miRNA signature performed for the COAD and READ TCGA CRC patients with different tumor stages revealed a potential prognostic value for the miRNAs hsa-miR-497-5p and hsa-miR-375-3p, whose expression levels increased (*p* = 0.0297) and decreased (*p* = 0.0372) significantly in more advanced tumors, respectively (Figure 12).

## 3. Discussion

Despite advances in screening, diagnostics, and treatment options, CRC remains one of the most prevalent and lethal cancers worldwide [1,30]. While early diagnosis and effective management strategies have improved, CRC is often diagnosed in a very advanced stage with a consequent poor prognosis. Therefore, identifying reliable biomarkers for early CRC diagnosis remains a critical challenge to improve and enhance the prognosis of patients.

In recent years, non-coding RNAs, particularly miRNAs, have garnered attention as potential diagnostic and prognostic biomarkers for different tumors, including CRC [12,26,27]. To clearly demonstrate the involvement of miRNAs in CRC, we investigated the expression levels of a set of miRNAs strongly associated with CRC development and previously identified using an analytical workflow developed at the Laboratory of Experimental Oncology at the University of Catania. In further detail, in our previous bioinformatic research, we identified a panel of miRNAs potentially involved in the development and progression of CRC by performing an integrated computational analysis, taking into account relevant miRNA expression datasets contained in the GEO DataSets databases [26]. Building upon this previous study, in the present manuscript, we tested the expression levels of the predicted upregulated miRNAs hsa-miR-21-5p and hsa-miR-503-5p, and the predicted downregulated miRNAs hsa-miR-497-5p and hsa-miR-375-3p on FFPE and liquid biopsy samples obtained from CRC patients and healthy donors, using the highly sensitive ddPCR platform.

The analysis of the expression levels of the selected miRNAs in the CRC FFPE tissue samples and normal adjacent mucosa revealed statistically significant higher levels of hsa-miR-21-5p and hsa-miR-503-5p and a significant reduction in hsa-miR-497-5p in tumor samples compared to controls. The diagnostic potential of these miRNAs was further confirmed by performing ROC analyses, which demonstrated that the two upregulated miRNAs in the FFPE samples were able to effectively diagnose CRC compared to non-malignant lesions.

In contrast, less robust results were obtained when liquid biopsy samples were analyzed. Indeed, none of the circulating miRNAs showed significant increments or decrements, but only a non-significant trend of variation was observed for the miRNAs hsa-miR-21-5p and hsa-miR-375.

Taken together, the ddPCR results and ROC analyses performed for the four selected miRNAs in FFPE samples suggested that hsa-miR-21-5p and hsa-miR-503-5p are valuable diagnostic biomarkers for CRC, whereas none of the selected miRNAs seem to be reliable predictors of CRC when evaluated in liquid biopsy samples. Confirming our results, other studies have already proposed hsa-miR-21-5p as a possible diagnostic biomarker for CRC [31,32]. Although some studies have also proposed hsa-miR-21-5p as a valid circulating biomarker [25,33], our results found no significant difference in the expression levels of this miRNA between the liquid biopsy samples of healthy subjects and CRC patients. Similarly, the results obtained for hsa-miR-497-5p were also confirmed in the literature, where it was found to be downregulated in CRC and its overexpression seemed to play a positive role in anticancer effects [34,35]. More scientific evidence was obtained for the downregulated hsa-miR-375-3p, which is recognized as a biomarker for CRC when downregulated [36,37].

After validating the diagnostic potential of the selected miRNAs, we were also interested in exploring their prognostic significance in predicting the survival of CRC patients, however, no follow-up data were collected for the patients recruited in the study. Therefore, computational analyses were performed, both to establish the functional roles of the four validated miRNAs and to verify their prognostic value in CRC.

The miRTargetLink Human analysis integrated with miRDip, TargetScan, and miRWalk allowed us to identify 17 genes modulated by the four selected miRNAs. Through the STRING and GO Panther analyses, we found that these 17 genes were able to interact with each other and thus regulate various molecular and biological processes. Notably, some of the 17 identified genes, such as PIK3R1 [38], SMAD7 [39], VEGFA [40], BCL2 [41], etc., are already known to be involved in the development and progression of CRC or other tumors when modulated by miRNAs.

To investigate the prognostic potential of the selected miRNAs, we consulted the OncoLnc tool to analyze the data contained in the TCGA COAD and READ databases. The results obtained from this analysis revealed that all the selected miRNAs could also have a prognostic role. Specifically, contrary to what was expected and what is usually reported in the literature, hsa-miR-21-5p seemed to correlate with better prognosis when overexpressed in the READ cohort while no significant difference was obtained from the survival data obtained from the COAD database. With regards to hsa-miR-503-5p and hsa-miR-497-5p, OS analyses did not provide significant data when considering the READ database, whereas from the COAD database, both appeared to have a possible prognostic value. In particular, the overexpression of hsa-miR-503-5p was associated with a poorer prognosis, while the downregulated hsa-miR-497-5p also correlates with a worse prognosis when highly expressed, as also reported by Zou G and colleagues [42]. Finally, hsa-miR-375-3p was strongly associated with a worse prognosis when it was poorly expressed in COAD patients. Indeed, both hsa-miR-375-3p downregulation and hsa-miR-497-5p dysregulation seemed to be associated with more advanced tumor stages, as recently demonstrated in other tumors [43,44]. Although these data could seem conflicting, several of the literature findings suggest different miRNA expression between left- and right-sided tumors or between rectum and colon cancer [45,46,47].

Although the results obtained were interesting, the present study presented some limitations. First, the lack of follow-up visits from the patients included and analyzed in this study did not allow us to effectively evaluate the prognostic significance of the proposed four-miRNA signature. Secondly, the FFPE and liquid biopsy samples were not coupled, therefore, the tissue expression of miRNAs could not be compared with the same circulating counterpart. Third, the case series included in this study was limited; however, the data obtained were also confirmed by some bioinformatics investigations and in our previous computational study performed on a virtual cohort of thousands of CRC cases, thus reducing the impact of this limitation. Overall, the results obtained here suggest the diagnostic and prognostic significance of this four-miRNA signature for CRC, encouraging further studies on a wider cohort of matched tissue and liquid biopsy samples. Particularly, more robust studies on tumor-circulating miRNAs are mandatory to prove the predictive role of miRNAs in non-invasive diagnostic procedures. For this purpose, it could be useful to evaluate circulating miRNA expression on circulating tumor cells or exosomes isolated from liquid biopsy samples [48].

## 4. Materials and Methods

### 4.1. Patient Cohorts and Specimens

For this study, a total of 20 CRC FFPE samples and matched adjacent normal colon mucosa were selected from the biobank of the Pathology Unit at the University of Catania. Liquid biopsy samples were further collected from 10 patients with a confirmed diagnosis of CRC and 10 healthy donors who were used as normal controls. Informed consent was obtained from all the patients and controls included in the study. From these patients, blood samples were collected and centrifugated at 2000× *g* for 10 min at room temperature to separate the serum from the blood cells. The aliquots thus obtained were then stored at −80 °C until their use. The socio-demographic and clinicopathological features of the patients included in this study are reported in Table 2.

### 4.2. RNA Extraction and microRNA Reverse Transcription

The extraction of miRNAs from the FFPE samples was performed using the miRNeasy FFPE kit (Qiagen—Cat. No. 217504, Hilden, Germany). In particular, total RNA, including miRNAs, was extracted from four tissue sections of 5–8 μm thickness for both tumor samples and adjacent normal mucosa as per the protocol.

Circulating total RNA was extracted from serum samples using the miRNeasy Serum/Plasma kit (Cat. No. 217184, Qiagen, Hilden, Germany). In particular, circulating miRNAs were extracted from 200 μL of serum using the manufacturer’s instructions and using the exogenous synthetic UniSp4 as a spike-in control (Cat. No. 339390, Qiagen, Hilden, Germany) to normalize the absolute quantification levels of the extracted circulating miRNAs. Finally, 4 μL of the extracted miRNAs was reverse transcribed into cDNA using the miRCURY LNA RT Kit (Cat. No. 339340, Qiagen, Hilden, Germany).

### 4.3. ddPCR for Absolute Quantification of microRNA Expression Levels

The quantification of miRNAs in both the FFPE and liquid biopsy samples was performed using the ddPCR assay. Qiagen LNA primers specific to the four selected miRNAs were adopted. In addition, specific primers were also used for the analysis of the abundance of the Unisp4 spike-in control (miRCURY LNA miRNA PCR Assays, Qiagen—Cat. No. 339306) and endogenous U6 snRNA, used as small RNA control for the quantification of the miRNA expression levels in the liquid biopsy and FFPE samples, respectively. Specifically, the ddPCR amplification reaction was prepared by using 11 µL of 2x QX200 ddPCR EvaGreen^®^ Supermix (Cat. No. 1864034—Bio-Rad, Hercules, CA, USA), 1.1 μL of the miRNA-specific primer miRCURY LNA miRNA PCR Assay (Cat. No. 339306 Qiagen, Hilden, Germany), 6.9 μL of RNase and DNase free-water, and 3 μL of cDNA for a total reaction volume of 22 μL. After the generation of the droplets, the samples were amplified according to the manufacturer’s protocol. After amplification, negative and positive droplets were read in the QX200 Droplet Reader (Bio-Rad, Hercules, CA, USA). Data analysis was performed using QuantaSoft Software version 1.7 (Bio-Rad).

### 4.4. Bioinformatics Analyses

In order to assess the correlation between the selected miRNAs and the clinicopathological features of patients affected by CRC, further bioinformatics analyses were performed. The bioinformatics software miRTargetLink Human, providing a comprehensive database for experimentally validated miRNA–target interactions considering their biological relevance, was consulted to identify the genes targeted by the selected miRNAs, while the software miRDip, TargetScan, and miRWalk, integrating multiple miRNA-target databases, were used to confirm and implement the missing data in miRTargetLink Human 2.0, if any. Subsequently, GO enrichment tools, including STRING (Search Tool for the Retrieval of Interacting Genes/Proteins) and GO Panther software (V 19.0), were used to evaluate the functional role of the genes targeted by the selected miRNAs. STRING was employed to investigate potential protein–protein interactions, which can provide insights into the broader biological pathways influenced by the genes, while GO Panther was utilized to classify genes based on their biological processes, molecular functions, and cellular components. These tools were chosen for their ability to uncover functional patterns and relationships, thereby enhancing the understanding of the biological significance of the genes targeted by the selected miRNAs. Finally, two other tools were used to analyze the clinical data contained in the TCGA COAD and TCGA READ databases. Particularly, the GEPIA (Gene Expression Profiling Interactive Analysis) portal was used to evaluate the dysregulation of the genes targeted by the four validated miRNAs, while OncoLnc was used to evaluate the prognostic significance of the selected miRNAs in the prediction of the overall survival of the COAD and READ patients. TCGA COAD and READ databases were also used to analyze the expression levels of the four-miRNA signature in different tumor stages to further validate their prognostic significance in CRC.

### 4.5. Statistical Analyses

The Kolmogorov–Smirnov normality test was used to assess the distribution of the expression levels of hsa-miR-21-5p, hsa-miR-497-5p, hsa-miR-503-5p, and hsa-miR-375; the Wilcoxon test was used to determine statistical differences between FFPE tumor samples and matched adjacent normal mucosa; and the Mann–Whitney test was used to conduct statistical analysis of the miRNA expression levels in liquid biopsy samples. The Kruskal–Wallis test was used for the analysis of miRNA expression levels in different tumor stages, taking the data collected in the TCGA COAD and READ databases. All the data are presented as mean ± standard error of the mean (SEM). The specificity and sensitivity of the analyzed miRNAs were assessed by performing receiver operating characteristic (ROC) curve analysis. All statistical analyses were conducted using GraphPad Prism v.8.

## 5. Conclusions

The results obtained in this study demonstrate the diagnostic potential of a four-miRNA signature significantly altered in CRC when analyzed in FFPE tissue samples, whereas their performance in liquid biopsy samples was less robust. Among the selected miRNAs, hsa-miR-21-5p, hsa-miR-503-5p, and hsa-miR-497-5p showed promising diagnostic potential in tissue samples, as confirmed by ddPCR and ROC analyses. Additionally, bioinformatic analyses revealed that these miRNAs may have prognostic significance, particularly hsa-miR-375-3p and hsa-miR-497-5p, given their association with key molecular pathways and genes implicated in CRC development and progression.

Despite these promising findings, limitations such as the lack of follow-up data and matched tissue–liquid biopsy samples underscore the need for further validation studies. Future research should focus on larger and more different cohorts, as well as paired tissue and liquid biopsy samples, to confirm and expand on these results. Ultimately, this four-miRNA signature holds potential as a dual-purpose biomarker panel for both the diagnosis and prognosis of CRC, offering a way to improve patient management and outcomes and to personalize treatments [49].

## Figures and Tables

**Table 1:** Sensitivity and specificity rates of the four selected miRNAs, according to ROC curve analysis.

miRNA	Sensitivity	Specificity
hsa-miR-21-5p	100%	80%
hsa-miR-503-5p	90%	70%
hsa-miR-375-3p	-	-
hsa-miR-497-5p	-	-

**Table 2:** Socio-demographic and clinicopathological features of the CRC patients and controls.

	FFPE		Liquid Biopsy				Fisher’s Test Chi-Square Test
	Cancer Patients (N. 10)		Cancer Patients (N. 10)		Normal Controls (N. 10)		
	N.	%	N.	%	N.	%	
Sex							
Male	5	50	7	70	9	90	0.5820
Female	5	50	3	30	1	10	
Age							
<60	0	0	2	20	6	60	0.1698
≥60	10	100	8	80	4	40	
Smoke							
Yes	NA		2	20	3	30	0.8646
No			6	60	5	50	
Ex-smoker			2	20	2	20	
Tumor Stage							
T1	0	0	1	10	NA		
T2	1	10	2	20			
T3	6	60	5	50			
T4	3	30	1	10			
Lymph Nodes							
Positive	5	50	5	50	NA		
Negative	4	40	5	50			
Nx	1	10	0	0			

NA: Not available.

## Data Availability

All data generated or analyzed during this study are included in this published article. All the data are available from the corresponding author on reasonable request.

## Supplementary Materials

The following supporting information can be downloaded at: https://www.mdpi.com/article/10.3390/ijms26031219/s1.

## Abbreviations

The following abbreviations are used in this manuscript:

CRC	Colorectal cancer
FOBT	Fecal occult blood test
FIT	Fecal immunochemical test
miRNA	microRNA
EMT	Epithelial-to-mesenchymal transition
FFPE	Formalin-fixed paraffin-embedded
ddPCR	droplet digital PCR
STRING	Search Tool Retrieval of Interacting Genes/Proteins
TCGA	The Cancer Genome Atlas
GEPIA	Gene Expression Profiling Interactive Analysis
ROC	Receiver Operating Characteristics

## References

[1] Filho A.M., Laversanne M., Ferlay J., Colombet M., Piñeros M., Znaor A., Parkin D.M., Soerjomataram I., Bray F. (2024). The GLOBOCAN 2022 cancer estimates: Data sources, methods, and a snapshot of the cancer burden worldwide. Int. J. Cancer.

[2] Blom J., Saraste D., Törnberg S., Jonsson H. (2024). Routine Fecal Occult Blood Screening and Colorectal Cancer Mortality in Sweden. JAMA Netw. Open.

[3] Zhu Y., Li X., Hu Y., Chen K., Zheng S., Ding K., Colorectal Cancer Screening Cohort Research Group (2023). Nonadherence to Referral Colonoscopy After Positive Fecal Immunochemical Test Results Increases the Risk of Distal Colorectal Cancer Mortality. Gastroenterology.

[4] Metaxas G., Papachristou A., Stathaki M. (2024). Colorectal cancer screening: Modalities and adherence. World J. Gastroenterol..

[5] Kildusiene I., Dulskas A., Smailyte G. (2024). Value of combined serum CEA, CA72-4, and CA19-9 marker detection in diagnosis of colorectal cancer. Tech. Coloproctol..

[6] Lakemeyer L., Sander S., Wittau M., Henne-Bruns D., Kornmann M., Lemke J. (2021). Diagnostic and Prognostic Value of CEA and CA19-9 in Colorectal Cancer. Diseases.

[7] Cao Q., Tian Y., Deng Z., Yang F., Chen E. (2024). Epigenetic Alteration in Colorectal Cancer: Potential Diagnostic and Prognostic Implications. Int. J. Mol. Sci..

[8] Wang Y., Wang C., Zhong R., Wang L., Sun L. (2024). Research progress of DNA methylation in colorectal cancer. Mol. Med. Rep..

[9] Qiu W., Akanyibah F.A., Xia Y., Ocansey D.K.W., Mao F., Liang Y. (2025). Emerging role of small RNAs in inflammatory bowel disease and associated colorectal cancer. Int. J. Mol. Med..

[10] Candido S., Lupo G., Pennisi M., Basile M.S., Anfuso C.D., Petralia M.C., Gattuso G., Vivarelli S., Spandidos D.A., Libra M. (2019). The analysis of miRNA expression profiling datasets reveals inverse microRNA patterns in glioblastoma and Alzheimer’s disease. Oncol. Rep..

[11] Gao X., Yang X., He F., Liu X., Liu D., Yuan X. (2023). Downregulation of microRNA 494 inhibits cell proliferation in lung squamous cell carcinoma via the induction of PUMA α mediated apoptosis. Exp. Ther. Med..

[12] Falzone L., Grimaldi M., Celentano E., Augustin L.S.A., Libra M. (2020). Identification of Modulated MicroRNAs Associated with Breast Cancer, Diet, and Physical Activity. Cancers.

[13] Falzone L., Lupo G., La Rosa G.R.M., Crimi S., Anfuso C.D., Salemi R., Rapisarda E., Libra M., Candido S. (2019). Identification of Novel MicroRNAs and Their Diagnostic and Prognostic Significance in Oral Cancer. Cancers.

[14] Stella M., Russo G.I., Leonardi R., Carcò D., Gattuso G., Falzone L., Ferrara C., Caponnetto A., Battaglia R., Libra M. (2024). Extracellular RNAs from Whole Urine to Distinguish Prostate Cancer from Benign Prostatic Hyperplasia. Int. J. Mol. Sci..

[15] Pekarek L., Sánchez Cedra A., Jaudenes Y.D.Y., Ospino L.R., Iglesias Pedrejón B., Bernier L., Roberts Cervantes E.D., Sánchez Cendra C., Cassinello J., Trasobares L. (2024). Paradigm of biomarkers in metastatic melanoma. Oncol. Lett..

[16] Ždralević M., Raonić J., Popovic N., Vučković L., Rovčanin Dragović I., Vukčević B., Todorović V., Vukmirović F., Marzano F., Tullo A. (2023). The role of miRNA in colorectal cancer diagnosis: A pilot study. Oncol. Lett..

[17] Gattuso G., Crimi S., Lavoro A., Rizzo R., Musumarra G., Gallo S., Facciponte F., Paratore S., Russo A., Bordonaro R. (2022). Liquid Biopsy and Circulating Biomarkers for the Diagnosis of Precancerous and Cancerous Oral Lesions. Noncoding RNA.

[18] Cui M., Wang H., Yao X., Zhang D., Xie Y., Cui R., Zhang X. (2019). Circulating MicroRNAs in Cancer: Potential and Challenge. Front. Genet..

[19] Pekarek L., Torres-Carranza D., Fraile-Martinez O., García-Montero C., Pekarek T., Saez M.A., Rueda-Correa F., Pimentel-Martinez C., Guijarro L.G., Diaz-Pedrero R. (2023). An Overview of the Role of MicroRNAs on Carcinogenesis: A Focus on Cell Cycle, Angiogenesis and Metastasis. Int. J. Mol. Sci..

[20] Bader El Din N.G., El Shenawy R., Moustafa R.I., Khairy A., Farouk S. (2024). Association between the expression level of miRNA 374a and TGF-beta;1 in patients with colorectal cancer. World Acad. Sci. J..

[21] Xie Y., Zhang Y., Liu X., Cao L., Han M., Wang C., Chen J., Zhang X. (2023). miR-151a-5p promotes the proliferation and metastasis of colorectal carcinoma cells by targeting AGMAT. Oncol. Rep..

[22] Pizzini S., Bisognin A., Mandruzzato S., Biasiolo M., Facciolli A., Perilli L., Rossi E., Esposito G., Rugge M., Pilati P. (2013). Impact of microRNAs on regulatory networks and pathways in human colorectal carcinogenesis and development of metastasis. BMC Genom..

[23] Gaedcke J., Grade M., Camps J., Søkilde R., Kaczkowski B., Schetter A.J., Difilippantonio M.J., Harris C.C., Ghadimi B.M., Møller S. (2012). The rectal cancer microRNAome--microRNA expression in rectal cancer and matched normal mucosa. Clin. Cancer Res..

[24] Hedayat S., Cascione L., Cunningham D., Schirripa M., Lampis A., Hahne J.C., Tunariu N., Hong S.P., Marchetti S., Khan K. (2024). Circulating microRNA Analysis in a Prospective Co-clinical Trial Identifies MIR652-3p as a Response Biomarker and Driver of Regorafenib Resistance Mechanisms in Colorectal Cancer. Clin. Cancer Res..

[25] AlZaabi A., Shalaby A. (2024). A Systematic Review of Diagnostic Performance of Circulating MicroRNAs in Colorectal Cancer Detection with a Focus on Early-Onset Colorectal Cancer. Int. J. Mol. Sci..

[26] Falzone L., Scola L., Zanghì A., Biondi A., Di Cataldo A., Libra M., Candido S. (2018). Integrated analysis of colorectal cancer microRNA datasets: Identification of microRNAs associated with tumor development. Aging.

[27] Crimi S., Falzone L., Gattuso G., Grillo C.M., Candido S., Bianchi A., Libra M. (2020). Droplet Digital PCR Analysis of Liquid Biopsy Samples Unveils the Diagnostic Role of hsa-miR-133a-3p and hsa-miR-375-3p in Oral Cancer. Biology.

[28] Miotto E., Saccenti E., Lupini L., Callegari E., Negrini M., Ferracin M. (2014). Quantification of circulating miRNAs by droplet digital PCR: Comparison of EvaGreen- and TaqMan-based chemistries. Cancer Epidemiol. Biomarkers Prev..

[29] Gahlawat A.W., Witte T., Sinn P., Schott S. (2023). Circulating cf-miRNA as a more appropriate surrogate liquid biopsy marker than cfDNA for ovarian cancer. Sci. Rep..

[30] Falzone L., Bordonaro R., Libra M. (2023). SnapShot: Cancer Chemotherapy. Cell.

[31] Li J., Chen H., Sun G., Zhang X., Ye H., Wang P. (2023). Role of miR-21 in the diagnosis of colorectal cancer: Meta-analysis and bioinformatics. Pathol. Res. Pract..

[32] Liu T., Liu D., Guan S., Dong M. (2017). Diagnostic role of circulating MiR-21 in colorectal cancer: A update meta-analysis. Ann. Med..

[33] Rattan Negi R., Rana S.V., Gupta V., Gupta R., Dhawan D.K. (2024). Evaluation of the Plasma Expression Levels of miR-21 and miR-145 as Potential Non-Invasive Biomarkers for Early Detection of Colorectal Cancer. Asian Pac. J. Cancer Prev..

[34] Xu Y., Chen J., Gao C., Zhu D., Xu X., Wu C., Jiang J. (2017). MicroRNA-497 inhibits tumor growth through targeting insulin receptor substrate 1 in colorectal cancer. Oncol. Lett..

[35] Wang L., Jiang C.F., Li D.M., Ge X., Shi Z.M., Li C.Y., Liu X., Yin Y., Zhen L., Liu L.Z. (2016). MicroRNA-497 inhibits tumor growth and increases chemosensitivity to 5-fluorouracil treatment by targeting KSR1. Oncotarget.

[36] Fonseca A., Ramalhete S.V., Mestre A., Pires das Neves R., Marreiros A., Castelo-Branco P., Roberto V.P. (2021). Identification of colorectal cancer associated biomarkers: An integrated analysis of miRNA expression. Aging.

[37] Xu L., Li M., Wang M., Yan D., Feng G., An G. (2014). The expression of microRNA-375 in plasma and tissue is matched in human colorectal cancer. BMC Cancer.

[38] Yan L.X., Liu Y.H., Xiang J.W., Wu Q.N., Xu L.B., Luo X.L., Zhu X.L., Liu C., Xu F.P., Luo D.L. (2016). PIK3R1 targeting by miR-21 suppresses tumor cell migration and invasion by reducing PI3K/AKT signaling and reversing EMT, and predicts clinical outcome of breast cancer. Int. J. Oncol..

[39] Tang J., Li X., Cheng T., Wu J. (2021). miR-21-5p/SMAD7 axis promotes the progress of lung cancer. Thorac. Cancer.

[40] Qiu Y., Yu H., Shi X., Xu K., Tang Q., Liang B., Hu S., Bao Y., Xu J., Cai J. (2016). microRNA-497 inhibits invasion and metastasis of colorectal cancer cells by targeting vascular endothelial growth factor-A. Cell Prolif..

[41] Zaharie F., Muresan M.S., Petrushev B., Berce C., Gafencu G.A., Selicean S., Jurj A., Cojocneanu-Petric R., Lisencu C.I., Pop L.A. (2015). Exosome-Carried microRNA-375 Inhibits Cell Progression and Dissemination via Bcl-2 Blocking in Colon Cancer. J. Gastrointestin. Liver Dis..

[42] Zou G., Wang R., Wang M. (2019). Clinical response and prognostic significance of serum miR-497 expression in colorectal cancer. Cancer Biomark..

[43] Zhang Z., Zhou Y., Liang S. (2024). Correlation Between miR-497-5p Expression with Clinicopathological Characteristics and Prognosis in Patients with Breast Cancer. Appl. Immunohistochem. Mol. Morphol..

[44] Gan J., Zhang Y., Liu S., Mu G., Zhao J., Jiang W., Li J., Li Q., Wu Y., Wang X. (2023). MicroRNA-375 restrains the progression of lung squamous cell carcinoma by modulating the ERK pathway via UBE3A-mediated DUSP1 degradation. Cell Death Discov..

[45] De Nunzio V., Donghia R., Pesole P.L., Coletta S., Calò N., Notarnicola M. (2023). Serum Cytokine and miRNA Levels Are Differently Expressed in Right- and Left-Sided Colon Cancer. J. Clin. Med..

[46] Slattery M.L., Herrick J.S., Pellatt D.F., Mullany L.E., Stevens J.R., Wolff E., Hoffman M.D., Wolff R.K., Samowitz W. (2016). Site-specific associations between miRNA expression and survival in colorectal cancer cases. Oncotarget.

[47] Slattery M.L., Wolff E., Hoffman M.D., Pellatt D.F., Milash B., Wolff R.K. (2011). MicroRNAs and colon and rectal cancer: Differential expression by tumor location and subtype. Genes Chromosomes Cancer.

[48] Min L., Zhu S., Chen L., Liu X., Wei R., Zhao L., Yang Y., Zhang Z., Kong G., Li P. (2019). Evaluation of circulating small extracellular vesicles derived miRNAs as biomarkers of early colon cancer: A comparison with plasma total miRNAs. J. Extracell. Vesicles.

[49] Marletta S., Rizzo A., Spoto G., Falzone L. (2024). Predictive and prognostic biomarkers in cancer: Towards the precision medicine era. Explor. Target Antitumor. Ther..

