# Supplementary material for: Diagnostic and Prognostic Significance of a Four-miRNA Signature in Colorectal Cancer
Source: Int J Mol Sci. 2025 Jan 30;26(3):1219. doi: 10.3390/ijms26031219 (PMC11818852; doi:10.3390/ijms26031219)
Supplement: Supplementary file 1 [file ijms-26-01219-s001.zip › ijms-3447698-supplementary.pdf]

Supplementary Table S1. Expression levels of miRNAs according to patients' sociodemographic and clinical-pathological characteristics.

	FFPE Cancer Patients (N. 10)		p-value FFPE analysis				Liquid Biopsy Cancer Patients (N. 10)		p-value Liquid Biopsy analysis			
	N.	%	miR-21-5p	miR-375-3p	miR-503-5p	miR-497-5p	N.	%	miR-21-5p	miR-375-3p	miR-503-5p	miR-497-5p
**Sex**												
Male	5	50	>0,9999	0,2222	0,8889	>0,9999	7	70	0,5167	0,5167	0,8333	0,6667
Female	5	50					3	30				
**Age**												
<60	0	0	/	/	/	/	2	20	0,8889	0,4000	0,7111	0,2667
>=60	10	100					8	80				
**Smoke**												
Yes	NA		/	/	/	/	2	20	0,8413	0,6984	0,0889	0,9111
No							6	60				
Ex-smoker							2	20				
**Tumor Stage**												
T1	0	0	0,6944	0,6238	0,2976	0,9214	1	10	0,2889	0,6677	0,3582	0,6566
T2	1	10					2	20				
T3	6	60					5	50				
T4	3	30					1	10				
**Lymph Nodes**												
Positive	5	50	0,1825	0,3460	0,0873	0,1825	5	50	>0,9999	0,2222	>0,9999	0,8413
Negative	4	40					5	50				
Nx	1	10					0	0				

Mann-Whitney test was performed for sex and age variables, while Kruskal-Wallis test was performed for smoke, tumor stage and lymph node status; NA: Not available
